# Development, administration, and validity evidence of a subspecialty preparatory test toward licensure: a pilot study

**DOI:** 10.1186/s12909-018-1294-z

**Published:** 2018-08-01

**Authors:** John Johnson, Alan Schwartz, Matthew Lineberry, Faisal Rehman, Yoon Soo Park

**Affiliations:** 1 London Health Sciences Centre-University Hospital, Western University, 339 Windermere Road, London, ON N6A 5A5 Canada; 2 Medical Education, University of Illinois at Chicago, Chicago, IL USA; 3 Medical Education, University of Kansas, Kansas City, KS USA; 4 Medical Education, University of Illinois at Chicago, Chicago, IL USA

**Keywords:** Assessment, Validity evidence, Messick’s framework, Constructed-response item, Formative test, Questionnaire

## Abstract

**Background:**

Trainees in medical subspecialties lack validated assessment scores that can be used to prepare for their licensing examination. This paper presents the development, administration, and validity evidence of a constructed-response preparatory test (CRPT) administered to meet the needs of nephrology trainees.

**Methods:**

Learning objectives from the licensing examination were used to develop a test blueprint for the preparatory test. Messick’s unified validity framework was used to gather validity evidence for content, response process, internal structure, relations to other variables, and consequences. Questionnaires were used to gather data on the trainees’ perception of examination preparedness, item clarity, and curriculum adequacy.

**Results:**

There were 10 trainees and 5 faculty volunteers who took the test. The majority of trainees passed the constructed-response preparatory test. However, many scored poorly on items assessing renal pathology and physiology knowledge. We gathered the following five sources of validity evidence: (1) Content: CRPT mapped to the licensing examination blueprint, with items demonstrating clarity and range of difficulty; (2) Response process: moderate rater agreement (intraclass correlation = .58); (3) Internal structure: sufficient reliability based on generalizability theory (G-coefficient = .76 and Φ-coefficient = .53); (4) Relations to other variables: CRPT scores reflected years of exposure in nephrology and clinical practice; (5) Consequences: post-assessment survey revealed that none of the test takers felt “poorly prepared” for the upcoming summative examination and that their studying would increase in duration and be adapted in terms of content focus.

**Conclusions:**

Preparatory tests using constructed response items mapped to licensure examination blueprint can be developed and used at local program settings to help prepare learners for subspecialty licensure examinations. The CRPT and questionnaire data identified shortcomings of the nephrology training program curriculum. Following the preparatory test, trainees expressed an improved sense of preparedness for their licensing examination.

## Background

Subspecialty training programs administer locally-developed assessments to prepare learners toward certification. In Canada, nephrology is classified as a medical subspecialty for which training entails a 2-year commitment completed during postgraduate years (PGY) four and five. Three months after successful completion of a nephrology training program, trainees complete a 3-h, 55-item short-answer constructed-response (CR) test administered by the Royal College of Physicians and Surgeons of Canada (RCPSC), the national governing body for medical and surgical specialty training programs. The summative RCPSC CR test is modelled after the medical expert competencies for Canadian nephrology program trainees and was constructed and updated by the College in 2012.

Unfortunately, there is no published literature on preparatory tests for the RCPSC licensing examination instituted by nephrology training programs in Canada. In contrast, the American Society of Nephrology In-Training Examination (ASN ITE) in the United States began administering a nationally-developed preparatory examination since 2009 [1]. The ASN ITE is a 150 multiple-choice question assessment covering the test blueprint and assessment format of the American Board of Internal Medicine’s nephrology certification examination. As such, nephrology postgraduate programs in Canada require preparatory assessments towards certification – however, developing preparatory tests can be difficult, particularly for subspecialties with learners at advanced stages of training or for subspecialty programs with only a small number of learners; moreover, the RCPSC examination is a CR test that needs consideration for rater training and scoring. A RCPSC certification examination created in 2014 for General Internal Medicine trainees demonstrated content validity evidence of the performance scores [2]. In this sense, developing a preparatory assessment that adheres to promoting resident readiness for the RCPSC, while demonstrating sufficient validity would be useful and beneficial for trainees in postgraduate training.

Learner assessment at the “knows” and “knows how” levels of Miller’s Pyramid can be accomplished through written tests [3]. The practice of studying and taking a test has been shown to enhance learning compared to studying alone (e.g. re-reading course material) [4]. This process, also known as test-enhanced learning, conceptualizes assessment as a learning tool, rather than solely as tests [5, 6].

Furthermore, it is unclear whether learners receive constructive test performance feedback or an opportunity to evaluate the examination process due to lack of validity evidence in these locally-developed assessments. Developing and administering a subspecialty preparatory examination that includes robust validity evidence is an important and timely topic that has challenged postgraduate programs.

Messick’s unified validity framework provides a systematic approach for seeking construct validity evidence. Messick’s framework identifies the sources of validity evidence required to support (or negate) the appropriateness in the use of its test scores. Construct validity represents a summary of the evidence for and consequences of score interpretation and application. Identifying and collecting validity evidence enables one to make more accurate inferences on the usefulness of test scores and whether the test achieves its intended aim. Messick’s unified validity framework was used to gather validity evidence, focusing on content, response process, internal structure, relations to other variables, and consequences [7–9].

In this study, we developed, administered, and collected validity evidence for a short-answer CR preparatory test (CRPT) for nephrology trainees. Items in the CRPT were mapped to the RCPSC blueprint and administered to all nephrology trainees (five PGY4 and five PGY5) in June 2016, after informed consent was obtained. The CRPT was also administered to five practicing nephrology consultants, to examine their response patterns in comparison to the trainees. A scoring rubric was created in advance of the test administration, and three nephrology-medical education-trained individuals graded each test independently. This project created an assessment instrument that is the same testing format as the RCPSC licensing examination and also provided valuable test performance feedback to the CRPT takers. The study also included an evaluation of the CRPT, in the form of two questionnaires to gauge trainees’ perception of the newly developed test and their preparedness for the RCPSC summative examination. Lastly, trainees’ CRPT performance was used to identify any curricular deficiencies in the training program.

## Methods

### Content

#### Test development

The RCPSC learning objectives encompass the following seven content domains in nephrology: kidney transplantation (KT), renal physiology (RPh), acute kidney injury (AKI), renal pathology (RPa), chronic kidney disease (CKD), peritoneal dialysis (PD), and hemodialysis (HD). A two-dimensional test blueprint was developed to categorize proportions of items by domain and Bloom’s taxonomy of cognitive domains [10].

#### Content review

Content relevance, representativeness, and technical quality are important elements that contribute to content validity [11]. Test item quality is a fundamental consideration in assessment [3]. Published guidelines for writing SR items were used by CR test developers, specifically recommendations pertaining to item content, style concerns, and stem construction [12–14]. All test items were reviewed for clarity and cultural sensitivity by a medical educator [15]. Additionally, trainees evaluated each test item individually for its clarity and level of difficulty.

### Response process

#### Rater training

The CRPT was scored by graders who were blinded to the test takers identity/PGY level. To enhance grader consensus and cross-calibration, all graders were trained together on how to accurately and consistently apply the scoring rubric [3].

#### Scoring and rater consistency

The response data were analyzed using the points assigned to each test item (e.g. 8/10) allowing for partial credit, followed by a re-analysis where 1 point was assigned to each correctly answered item. Intraclass correlation (ICC) was used to assess the reliability of rater data.

### Internal structure

In addition to descriptive statistics, item characteristics (e.g. item difficulty) were examined. A high reliability indicates consistency in individual test takers’ scores [3]. Generalizability theory was used to evaluate the overall reliability of the CRPT and its variance components using a fully-crossed design, taking into account the overall effects of raters and items, candidates (*p*) x raters (*r*) x items (*i*).

### Relations to other variables

CRPT scores and response patterns were reviewed for different training levels of test takers and practicing nephrologists, hypothesizing learners with more advanced training would have higher performance.

### Consequences

#### Standard setting and pass rates

The consequential element of construct validity involves appraising both the short- and long-term effects of a test. Scores from a valid assessment instrument have the potential to improve both learning and instruction [7, 16]. An item-based standard setting method using the Extended Angoff procedure was used to set a passing score for the CRPT [3, 17].

#### Trainee perceptions of CRPT

The short-term impact of the CRPT was revealed by the questionnaire completed by trainees both before and following the formative examination. The long-term effects of the CRPT were measured by examining the changes recommended to the nephrology training program curriculum.

The Western University Non-Medical Research Ethics board approved the study.

## Results

### Content

Validity evidence supporting content indicated clarity in CRPT that all participating trainees (*n* = 10) rated 46 of the 55 items (84%) to be clearly written (Table 1). All of the nephrology trainees rated 16 of the 55 items (29%) as “not difficult” or “somewhat difficult”, and all of the test takers found 9 of the 55 items (16%) to be either “difficult”, “more difficult”, or “very difficult” (Table 1).

**Table 1:** Examinee perception of item clarity and level of difficulty

Item	Clarity (% clear)	Level of difficulty^a^ (% reporting)	Item	Clarity (% clear)	Level of difficulty^a^ (% reporting)
1	100	1 (80); 2 (20)	29	100	1 (20); 2 (20); 3 (30); 4 (30)
2	100	1 (20); 2 (40); 3 (40)	30	100	1 (80); 2 (20)
3	100	1 (40); 2 (40); 3 (20)	31	100	1 (10); 2 (60); 3 (40)
4	100	1 (50); 2 (50)	32	100	1 (10); 2 (10); 3 (30); 4 (30); 5 (20)
5	100	1 (40); 2 (60)	33	100	1 (90); 2 (10)
6	100	2 (20); 3 (60); 4 (20)	34	100	2 (100)
7	100	1 (20); 2 (80)	35	60	2 (30); 3 (40); 4 (30)
8	90	3 (60); 4 (30); 5 (10)	36	100	1 (30); 2 (50); 3 (20)
9	80	2 (40); 3 (50); 4 (10)	37	100	1 (50); 2 (40); 4 (10)
10	100	1 (20); 2 (80)	38	100	2 (50); 4 (50)
11	100	3 (20); 4 (70); 5 (10)	39	70	1 (30); 2 (40); 3 (30)
12	80	1 (30); 2 (50); 3 (20)	40	100	1 (90); 2 (10)
13	100	1 (20); 2 (80)	41	100	2 (30); 3 (70)
14	100	1 (20); 2 (50); 3 (30)	42	100	3 (20); 4 (50); 5 (30)
15	70	2 (50); 3 (50)	43	100	1 (60); 2 (40)
16	100	1 (40); 2 (60)	44	100	1 (70); 2 (30)
17	100	1 (80); 2 (20)	45	100	3 (40); 4 (40); 5 (20)
18	100	1 (10); 2 (50); 3 (40)	46	80	1 (70); 2 (30)
19	100	2 (30); 3 (70)	47	100	1 (70); 2 (20); 3 (10)
20	100	3 (40); 4 (60)	48	60	1 (20); 2 (20); 3 (60)
21	100	1 (20); 2 (30); 3 (40); 4 (10)	49	50	2 (20); 3 (60); 4 (20)
22	100	1 (10); 3 (30); 4 (60)	50	100	1 (80); 2 (20)
23	100	2 (30); 3 (40); 4 (30)	51	100	2 (5); 4 (50)
24	100	3 (10); 4 (50); 5 (40)	52	100	2 (10); 3 (50); 4 (40)
25	100	3 (20); 4 (60); 5 (20)	53	100	2 (60); 3 (20); 4 (20)
26	100	2 (30); 3(70)	54	100	1 (10); 2 (50); 3 (40)
27	100	2 (30); 3 (50); 4 (20)	55	100	1 (20); 2 (80)
28	100	3 (50); 4 (50)			

^a^Difficulty scale: 1, not difficult; 2, somewhat difficult; 3, difficult; 4, more difficult; 5, very difficult

### Response process

With a partial point marking scheme, the CRPT nephrology trainee (PGY4 and PGY5) score ranges for Graders 1, 2, and 3 were 51–68%, 49–68%, and 54–73% (Table 2). The inter-rater reliability of the scores indicated moderate agreement, ICC = 0.55; 95% CI = 0.09, 0.85 [18]. Overall, the standard deviation (SD) of the CRPT nephrology trainee scores (all Graders) was 4.33 points.

**Table 2:** Distribution of constructed response preparatory test nephrology trainees scores by grader (total potential points 261)

Test taker	Grader 1	Grader 2	Grader 3
*PGY4*			
1	51%	49%	54%
2	62%	57%	67%
3	65%	59%	69%
4	66%	62%	70%
5	68%	63%	73%
*PGY5*			
6	58%	63%	67%
7	62%	68%	66%
8	63%	67%	72%
9	63%	62%	70%
10	64%	68%	70%

The CRPT nephrology trainee scores were stratified into the seven content domains outlined in the test blueprint (Table 3). The highest and lowest test scores were in the domains of peritoneal dialysis and renal pathology, respectively.

**Table 3:** Distribution of constructed response preparatory test nephrology trainees scores breakdown by content domain

Test taker	Kidney transplant	Renal physiology	Acute kidney injury	Renal pathology	Chronic kidney disease	Peritoneal dialysis	Hemo-dialysis
*PGY4*							
1	50%	32%	60%	36%	46%	85%	71%
2	58%	59%	59%	18%	69%	100%	71%
3	75%	63%	57%	18%	63%	100%	75%
4	67%	52%	63%	18%	83%	100%	71%
5	77%	62%	63%	18%	71%	100%	79%
*PGY5*							
6	73%	46%	54%	36%	54%	77%	71%
7	58%	65%	63%	45%	77%	77%	38%
8	75%	54%	57%	27%	60%	92%	83%
9	67%	56%	62%	36%	63%	100%	67%
10	65%	49%	63%	45%	63%	85%	75%
Mean	67%	54%	60%	30%	65%	92%	70%

With a 1 point per correct item marking scheme, the CRPT nephrology trainee (PGY4 and PGY5) score ranges for Graders 1, 2, and 3 were 51–78%, 45–76%, and 55–79%. The inter-rater reliability of the scores indicated moderate agreement, ICC = 0.58; 95% CI = 0.16, 0.86. [18]

### Internal structure

Fifty-three percent of the test items were found to have an item-total correlation exceeding 0.20, indicating that items were able to discriminate between high and low performing test takers. Using a partial point marking scheme, the G-coefficient = 0.76 (normative uses of test scores; e.g., ranking learners), and the Φ-coefficient = 0.53 (criterion-based uses of test scores; e.g. pass-fail decisions). The largest variance was found in items (29.6%), indicating variability in item difficulty. Learner performance varied by item, which means some learners do well in some items while performing poorly on other items. Using a 1 point per correct item marking scheme, the G-coefficient = 0.60, and the Φ-coefficient = 0.48. The largest variance was again found in items (17%), and learner performance continued to vary by item.

### Relations to other variables

By PGY level, the CRPT scores were as follows: Grader 1, 51–68% for PGY4, and 58–64% for PGY5; Grader 2, 49–63% for PGY4, and 62–68% for PGY5; and Grader 3 54–73% for PGY4 and 66–72% for PGY5. By PGY level, the SDs of the CRPT score means (all Graders) were 5.90 for PGY4 and 5.33 for PGY5.

The CRPT nephrology trainee mean scores by content domains and PGY level were very similar to overall test scores (PGY4 and PGY5) divided by content domains alone (Tables 4 and 5).

**Table 4:** Distribution of constructed response preparatory test postgraduate year 4 nephrology trainee score breakdown by content domain

Test taker	Kidney transplant	Renal physiology	Acute kidney injury	Renal pathology	Chronic kidney disease	Peritoneal dialysis	Hemo- dialysis
1	50%	32%	60%	36%	46%	85%	71%
2	58%	59%	59%	18%	69%	100%	71%
3	75%	63%	57%	18%	63%	100%	75%
4	67%	52%	63%	18%	83%	100%	71%
5	77%	62%	63%	18%	71%	100%	79%
Mean	65%	54%	60%	22%	66%	97%	73%

**Table 5:** Distribution of constructed response preparatory test postgraduate year 5 nephrology trainee score breakdown by content domain

Test taker	Kidney transplant	Renal physiology	Acute kidney injury	Renal pathology	Chronic kidney disease	Peritoneal dialysis	Hemo-dialysis
1	73%	46%	54%	36%	54%	77%	71%
2	58%	65%	63%	45%	77%	77%	38%
3	75%	54%	57%	27%	60%	92%	83%
4	67%	56%	62%	36%	63%	100%	67%
5	65%	49%	63%	45%	63%	85%	75%
Mean	68%	54%	60%	38%	63%	86%	67%

The CRPT scores for nephrology consultants (*N* = 5) ranged from 76 to 83%, which were higher than the range of nephrology trainees, *p* = 0.48. The SD of the nephrology consultants’ CRPT mean scores was 2.42. Their highest and lowest mean content domain scores were in hemodialysis and renal pathology, respectively.

### Consequences

The CRPT raw passing score threshold was determined to be 57% based on application of the Extended Angoff procedure to all test graders. With that threshold, one nephrology trainee (PGY4) did not pass the CRPT.

Analysis of the results of the questionnaire distributed before the CRPT revealed that 8 of the 10 nephrology trainees felt “poorly prepared” or “not well prepared.” The questionnaire distributed after the CRPT revealed that none of the test takers felt “poorly prepared” for the RCPSC examination. For study habits post-CRPT, all 10 test takers felt their studying would increase in duration and be adapted in terms of content focus.

## Discussion

The development, administration, and collection of validity evidence for the CRPT provide meaningful information that can provide feedback to learners and the program to help prepare nephrology trainees for the licensure examination. Postgraduate trainees at advanced stages of training do not necessarily have preparatory assessments that provide meaningful information prior to taking the licensure examination. As such, this study provides a description on the test development and administration process as well as validity evidence supporting the use of the CRPT, following Messick’s unified validity framework.

Validity evidence supporting the CRPT is summarized as follows:

1. Content: 84% of the participants rated the items to be clearly written. The majority of questions on the CRPT have an appropriate level of difficulty based on item difficulty values. The majority of the items on the CRPT are able to discriminate between trainees with high and low test scores.
2. Response process: inter-rater reliability of the scores indicated moderate agreement. Acceptable inter-rater reliability may be the result of thorough rater training based on a scoring rubric prior to grading the test, using graders with a similar clinical background, and having graders with formal training in medical education.
3. Internal structure: 53% of the test items were found to have item discrimination exceeding 0.20, which indicates that items were able to discriminate between high and low performing trainees. Moreover, reliability based on generalizability theory indicate consistency in rank ordering trainees (G-coefficient = .76) and a level of confidence in making decisions (Φ-coefficient = .53).
4. Relations to other variables: CRPT scores were significantly higher for practicing nephrologists, when compared to trainees, reflecting differences in performance by years of exposure in nephrology and clinical practice.
5. Consequences: the questionnaire distributed after the test revealed that none of the test takers felt “poorly prepared” for the upcoming summative examination and that their studying would increase in duration and be adapted in terms of content focus.

Analysis of performance by content domain revealed strengths and weaknesses of both the trainees and training program. Possible reasons to explain the discrepancy in test scores across content domains include trainee rotations (and thus content exposure), varying degrees of item difficulty, and consultant knowledge base and expertise. The average degrees of item difficulty for questions pertaining to renal physiology and hemodialysis were 0.62 and 0.80, respectively. Like the trainees, the consultants scored lowest on items testing their knowledge of renal pathology and renal physiology.

The questionnaire distributed prior to taking the CRPT revealed the vast majority of trainees did not feel well prepared for the upcoming licensing examination. The questionnaire administered following the CRPT highlights the opportunity to apply one’s knowledge base during a three-hour test seems responsible for instigating the change in attitude towards exam readiness.

The limitations of the project include a small sample size, unavailable RCPSC nephrology licensure test scores, and a limited number of test items. A sample size of 10 limits the generalizability of our findings and increases the variability of the summary statistics. However, most subspecialties face challenges in acquiring adequate sample size. In fact, between 2014 and 2016, the mean number of RCPSC nephrology examinees per test administration is 19.3 [19]. Without RCPSC summative test scores available, it is challenging to demonstrate whether the CRPT actually predicts nephrology licensure performance. Moreover, the CRPT was modelled after the RCPSC licensing examination with 55 items, a 3-h time allowance, and assesses a limited aspect of the medical knowledge base required for a successful practice of nephrology.

## Conclusions

Validity evidence enables inferences to be made regarding the usefulness of test scores and whether a test achieves its intended aim. This study shows that a preparatory assessment developed in a local program can help prepare postgraduate trainees for licensure examination. We employed Messick’s unified validity framework to demonstrate the validity evidence gathered, which provides a systematic approach based on five sources of evidence. Validity evidence collected for the CRPT provides useful information regarding the utility of the CRPT as a training tool. The CRPT assesses a trainees’ competence in seven content domains, which may prove useful in the upcoming era of competency-based medical education. We also confirmed that we can use CRPT data to collect feedback about the adequacy of our training program curriculum, which can serve to guide curricular revisions.

## Abbreviations

AKI: Acute kidney injury
CKD: Chronic kidney disease
CR: Constructed-response
CRPT: Constructed-response preparatory test
HD: Hemodialysis
ICC: Intraclass correlation
KT: Kidney transplantation
PD: Peritoneal dialysis
PGY: Postgraduate year
RCPSC: Royal College of Physicians and Surgeons of Canada
RPa: Renal pathology
RPh: Renal physiology
SD: Standard deviation
SR: Selected-response
